# Genetic regulation of mouse liver metabolite levels

**DOI:** 10.15252/msb.20135004

**Published:** 2014-05-23

**Authors:** Anatole Ghazalpour, Brian J Bennett, Diana Shih, Nam Che, Luz Orozco, Calvin Pan, Raffi Hagopian, Aiqing He, Paul Kayne, Wen‐pin Yang, Todd Kirchgessner, Aldons J Lusis

**Affiliations:** ^1^ Division of CardiologyDepartment of MedicineUCLALos AngelesCAUSA; ^2^ Department of Molecular Cell and Developmental BiologyUCLALos AngelesCAUSA; ^3^ Department of Human GeneticsUCLALos AngelesCAUSA; ^4^ Department of Applied GenomicsBristol‐Myers SquibbPrincetonNJUSA; ^5^ Department of Atherosclerosis Drug DiscoveryBristol‐Myers SquibbPrincetonNJUSA; ^6^ Department of GeneticsUniversity of North Carolina at Chapel HillKannapolisNCUSA

**Keywords:** genome‐wide association, local eQTL, metabolome, QTL, transcriptome

## Abstract

We profiled and analyzed 283 metabolites representing eight major classes of molecules including Lipids, Carbohydrates, Amino Acids, Peptides, Xenobiotics, Vitamins and Cofactors, Energy Metabolism, and Nucleotides in mouse liver of 104 inbred and recombinant inbred strains. We find that metabolites exhibit a wide range of variation, as has been previously observed with metabolites in blood serum. Using genome‐wide association analysis, we mapped 40% of the quantified metabolites to at least one locus in the genome and for 75% of the loci mapped we identified at least one candidate gene by local expression QTL analysis of the transcripts. Moreover, we validated 2 of 3 of the significant loci examined by adenoviral overexpression of the genes in mice. In our GWAS results, we find that at significant loci the peak markers explained on average between 20 and 40% of variation in the metabolites. Moreover, 39% of loci found to be regulating liver metabolites in mice were also found in human GWAS results for serum metabolites, providing support for similarity in genetic regulation of metabolites between mice and human. We also integrated the metabolomic data with transcriptomic and clinical phenotypic data to evaluate the extent of co‐variation across various biological scales.

## Introduction

Metabolites are produced in the cell as a result of various enzymatic reactions and in part reflect the metabolic state of the cell (Sabatine *et al*, 2005; Sreekumar *et al*, 2009). Recent advances in high throughput technologies such as mass spectrometry and nuclear magnetic resonance have allowed investigators and clinicians to comprehensively measure and quantify such molecules with reasonable precision in tissues (Ellis *et al*, 2007; Wang *et al*, 2010). Investigators have begun to utilize these technologies to explore the relationships between metabolite levels and disease‐related traits for the identification of biomarkers and elucidation of mechanisms underlying disease (Newgard *et al*, 2009). These initial reports, primarily involving metabolites measured in the human blood serum, have shown that some metabolite levels are associated with complex disease phenotypes and may be used to assess risk factors for complex diseases such as diabetes (Wang *et al*, 2011), and frequently exhibit large heritability (Shah *et al*, 2009; Nicholson *et al*, 2011) and genetic variation due to only a handful of loci in the genome (Gieger *et al*, 2008; Illig *et al*, 2010; Suhre *et al*, 2011; Wang *et al*, 2011). In addition, efforts have begun to integrate metabolomic data with other molecular phenotypes such as transcriptomes and proteomes with the goal of elucidating the relevant pathways underlying complex disease phenotypes (Ferrara *et al*, 2008; Connor *et al*, 2010; Inouye *et al*, 2010).

The goal of the current study was to characterize the genetic landscape of liver metabolites in rodents. We quantified and performed genome‐wide association analysis (GWAS) of the liver metabolome in the Hybrid Mouse Diversity Panel (HMDP) comprised of 104 classical inbred strains and recombinant inbred strains (Bennett *et al*, 2010). While human metabolomic studies are largely restricted to either blood serum or urine, using experimental organism such as the mouse, it is possible to examine other tissues. Moreover, since the HMDP strains are inbred (and, therefore, renewable), metabolite levels can be readily integrated with other measured traits and molecular phenotypes. In the present study, we examined the extent of genetic regulation of metabolite levels in the liver of HMDP strains. These strains have been densely genotyped and, in most cases, fully sequenced and, thus, following correction of population structure, we were able to finely map loci controlling a large fraction of the measured metabolites. We also examined the extent of liver metabolites covariation with both liver transcriptomic data and 58 cardio‐metabolic traits.

## Results

### Metabolite profiling

**Figure 1:**
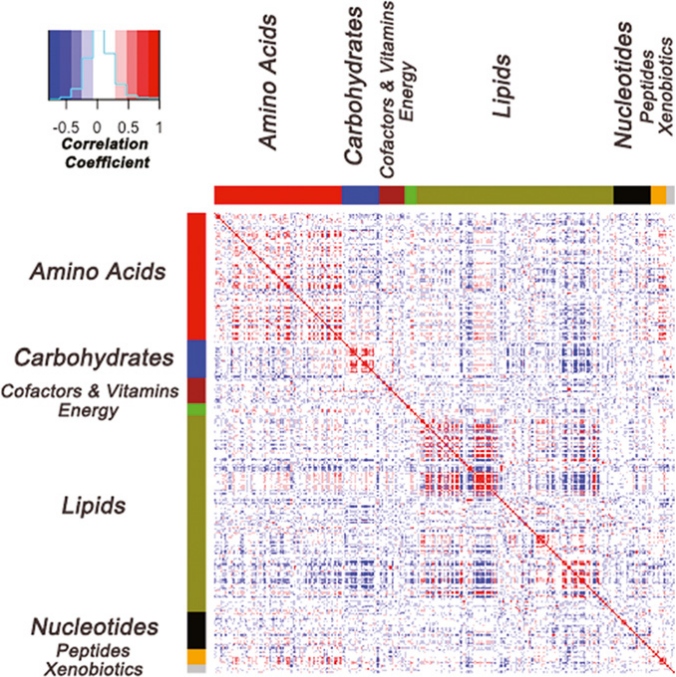
Correlation heatmap for the 283 metabolites measured in HMDP mouse liver The color bars on the top and the side of the heatmap represent the different classes as labeled. Within the heatmap, red represents a positive correlation, blue represents a negative correlation, and white represents a non‐significant correlation.

Using mass spectrometry (Metabolon Inc., Durham, NC) we examined liver metabolites in male mice across 104 inbred strains comprising the HMDP (for details of metabolite profiling see Materials and Methods). This methodology enabled us to quantify a comprehensive set of 283 metabolites present in mouse liver tissues across the HMDP panel. This set comprised of a diverse group of small molecules derived from the metabolism of four major class of macromolecules (Lipids, Carbohydrates, Proteins, Nucleic acids) as well as vitamins and cofactors, xenobiotics, and molecules related to energy metabolism. The transformed metabolite measurements can be found in Supplementary Table S1. We classified the 283 metabolites according to the primary metabolic pathway from which they are derived. The major classes identified were: lipid metabolism (“Lipids”), amino acid metabolism (“Amino Acids”), carbohydrate metabolism (“Carbohydrates”), nucleotide metabolism (“Nucleotides”), peptide‐derived molecules (“Peptides”), xenobiotics (“Xenobiotics”), vitamins and cofactors (“Cofactors”), and energy metabolism (“Energy”). From the 283 metabolites, the Lipids class represented the highest fraction (121 metabolites) followed by Amino Acids (78), Carbohydrates and Nucleotides (23 each), Peptides (10), Energy (7), and Xenobiotics (5) (Supplementary Fig S1). The metabolites exhibited significant correlations both within and between classes (Fig 1 and Supplementary Table S2). Overall, 1,639 pairwise correlations were significant after Bonferoni correction for multiple comparison (*P*‐value < 1.25e‐06), from which 68% (1,112/1,639) belonged to the within class pairs and 32% (527/1,639) belonged to between class correlations across all classes (Supplementary Table S2) reflecting shared biochemical pathways or regulatory interactions for metabolites across different classifications. We also observed that the extent to which metabolites correlated with other metabolites depended on the classification of the metabolites. Metabolites classified as “Cofactors and Vitamins”, “Energy”, and “Xenobiotics” exhibited the least and metabolites classified as “Lipids”, “Amino Acids”, and “Carbohydrates” had the largest number of significant within and between class correlations.

### Genetic regulation of metabolites

**Figure 2:**
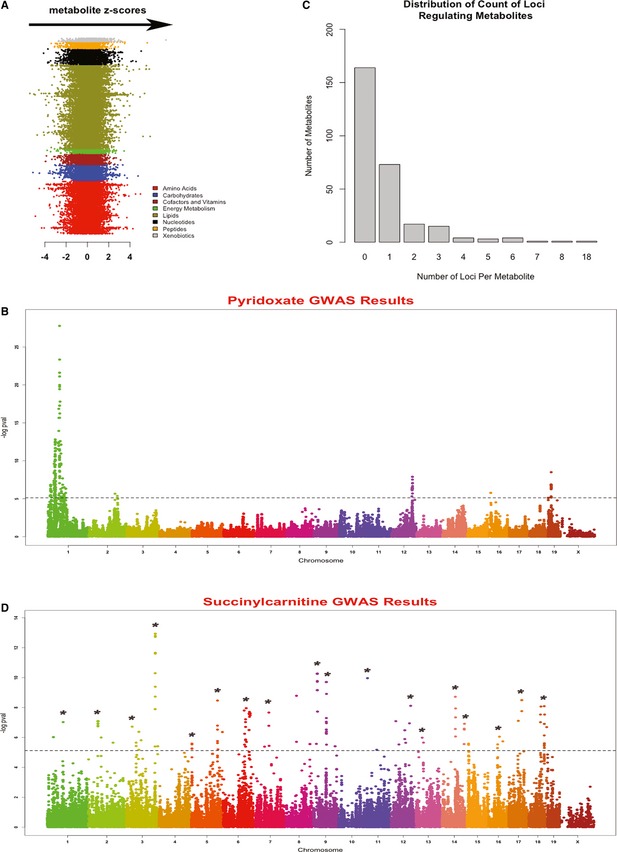
Summary of GWAS results for metabolites Metabolite variation in the HMDP mice. The *Z*‐score for each metabolite across the 104 HMDP mice is plotted along the *X*‐axis. Each row represents one of the 283 metabolites measured. Each metabolite is represented by the color shown in the color key.Genome‐wide plot for the most significant GWAS result (pyridoxate metabolite). The dotted line depicts the genome‐wide significance threshold.Distribution of the number of loci regulating metabolites.GWAS results for succinylcarnitine, which mapped to 18 distinct loci across the genome. The dotted line depicts the genome‐wide significance threshold. The asterisks depict the significant loci.

Figure 2A shows the variation of each of the 283 metabolites across the HMDP mice (each metabolite is ordered by its *z*‐score in the HMDP population). A high degree of variability, as evident by the wide distribution of *z*‐scores across all the eight major classes, reflects the influence of both genetic and environmental factors regulating metabolite levels. In order to explore the extent of genetic controls of metabolite variation, we compared the variance across the HMDP mice to the variance among five biological replicates in the commonly used C57BL/6J (B6) strain. Both the mean and median of variance for B6 mice were smaller than the entire population (mean of 0.36 in B6 versus 0.51 in HMDP, and median variance of 0.12 versus 0.30 in HMDP) suggesting the presence of genetic factors affecting metabolite variation in HMDP.

To identify the loci regulating the metabolite variation, we applied the genome‐wide association analysis approach utilizing 107,145 SNPs from the Broad Institute and Wellcome Trust Center database (http://www.broadinstitute.org/mouse/hapmap) which had a minor allele frequency >5% in the HMDP population of 104 inbred and recombinant inbred mouse strains. With this panel of SNPs, we performed genome‐wide association for each of the 283 metabolites using Efficient Mixed Modeling Algorithm (EMMA) that corrects for population structure and genetic relatedness among strains (Kang *et al*, 2008b). To avoid false positive associations resulting from over 20 million statistical tests performed in the GWAS, we set the statistical significance threshold at the genome‐wide FDR cutoff of 5% corresponding to the association *P*‐value of 7.6 × 10^−6^ (Storey & Tibshirani, 2003). This corresponds roughly to the genome‐wide significance level obtained using simulation analyses (Bennett *et al*, 2010). Significant linkage disequilibrium blocks occur among the HMDP strains (Frazer *et al*, 2007; Keane *et al*, 2011) and in such blocks (defined as SNPs exhibiting an *R*‐squared > 0.5), we only consider the peak SNP (i.e. the most significant SNP) as the true association.

**Figure 3:**
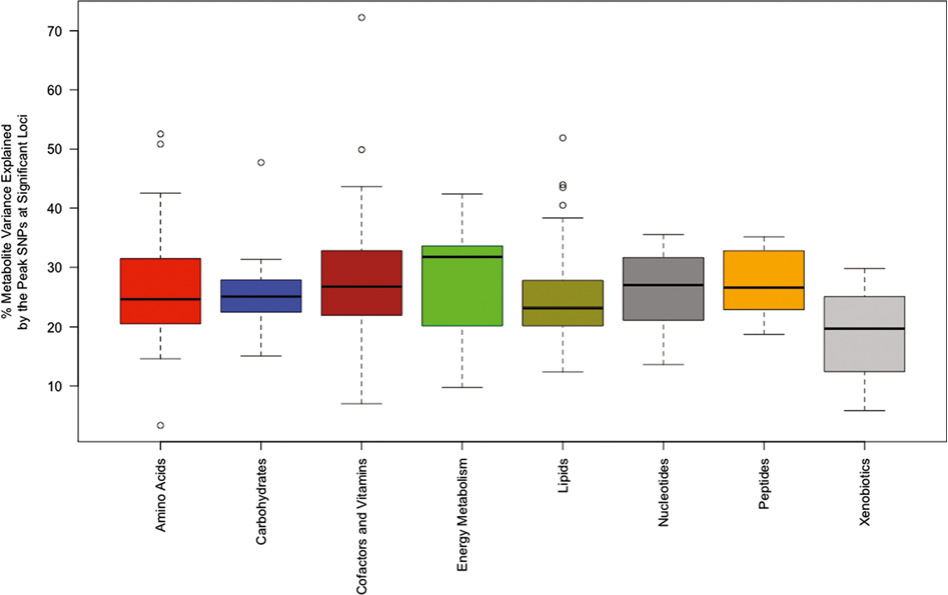
Genetic regulation of metabolite variation Comparison of percent metabolite variance explained by the peak maker in the GWAS analysis across various classes of metabolites. The largest variance explained was observed for the Vitamins and Cofactors class with the peak marker explaining 72% of the pyridoxate variation.

Using 5% FDR as the genome‐wide cutoff (*P*‐value = 7.6 × 10^−6^), we were able to identify 240 significant associations, from which 12 are expected to be false positives, for 119 of the 283 metabolites (Table 1 lists the top 10 associations and Supplementary Table S3 lists all the significant associations). Based on the peak SNP, the 240 significant associations corresponded to 227 distinct loci across the genome. The strongest association was found for pyridoxate mapping to the locus at 58.2 Mb on chromosome 1 with the *P*‐value of 1.6e‐28 (Fig 2B). The largest number of genome‐wide significant associations found at a single marker was on chromosome 19 at 46.7 Mb, corresponding to three metabolites (methylmalonate, malonate, and gulono‐1,4‐lactone). Defining the size of the locus as a 2 Mb window (1 Mb on each side of the peak SNP), there were 45 candidate genes residing at this locus including *Elovl3* (*3‐keto acyl‐CoA synthase*) which functions in the fatty acid elongation pathway. From the 119 metabolites with at least one significant association, 73 metabolites mapped to only one locus and the remaining 46 metabolites mapped to more than one locus (Fig 2C). The largest number of loci identified for any metabolite was for succinylcarnitine which mapped to more than 10 loci in the genome (Fig 2D). Calculation of percent variance explained for the peak markers underlying the 227 loci revealed that on average each significant peak SNP explained 26% of variation (Fig 3). We also found four loci that explained more than 50% of metabolite variations: the locus on chromosome 1 at 58.2 Mb explains 72% of pyridoxate variation, the locus on chromosome 7 at 152.4 Mb explains 52% of gamma‐aminobutyrate (GABA) variation, the locus on chromosome 8 at 119 Mb explains 51% of malonate variation, and the locus on chromosome 9 at 106.5 Mb explains 50% of N‐acetylglutamate variation.

**Table 1:** Top 10 GWAS results

Metabolite	Class	Pathways	GWAS *P*‐value	Chromosome	Base pair
Pyridoxate	Cofactors and vitamins	Vitamin B6 metabolism	1.66E‐28	1	58,236,240
Glucarate (saccharate)	Cofactors and vitamins	Ascorbate and aldarate metabolism	7.84E‐17	4	118,382,970
N‐acetylglutamate	Amino acids	Glutamate metabolism	1.09E‐15	9	106,563,342
Malonate (propanedioate)	Lipids	Mevalonate metabolism	2.59E‐15	8	119,096,872
Glycerate	Carbohydrates	Glycolysis, gluconeogenesis, pyruvate metabolism	2.11E‐14	12	43,670,942
Methylmalonate (MMA)	Amino acids	Valine, leucine and isoleucine metabolism	6.50E‐14	8	119,096,872
Succinylcarnitine	Energy	Krebs cycle	1.16E‐13	3	144,100,853
Methylmalonate (MMA)	Amino acids	Valine, leucine and isoleucine metabolism	4.79E‐13	19	46,764,525
Methylmalonate (MMA)	Amino acids	Valine, leucine and isoleucine metabolism	8.84E‐13	1	32,385,974
Kynurenate	Amino acids	Tryptophan metabolism	3.83E‐11	2	134,733,767

To assess the genetic complexity of metabolites in each class, we evaluated both the strength of the association *P*‐value and the percent variance explained by the significant loci identified and found no significant differences between the eight major classes (Fig 3 and Supplementary Fig S2). Using Fisher's Exact Test, we also found no over‐ or under‐representation of eight major classes of metabolites among the 119 loci mapped.

### Candidate gene prioritization

The number of candidate genes influencing metabolite levels at each associated locus varies from a few to dozens, depending upon the level of linkage disequilibrium. To help narrow down the list of candidate genes and to gain further insight into the molecular mechanism regulating metabolite levels, we integrated data from both the public database and from gene expression microarray analyses with the metabolite data. For the latter, we have previously reported the transcriptome profiling of the HMDP mice (Bennett *et al*, 2010) using Affymetrix HT_MG‐430A gene expression microarray platform consisting of over 23 thousand probes, as well as mapping of transcript levels using EMMA(Ghazalpour *et al*, 2011).

To identify potential candidate genes affecting metabolite levels, we took two different approaches: First we reasoned that if any enzyme or a gene is involved in the production or turnover of a metabolite within the cell, it will be functionally linked to the metabolite and both qualify as a potential candidate gene and validate the identified locus as a true association based on prior knowledge. For this, we examined public databases for biochemical pathways and, for each metabolite, we asked if any of the genes that are physically located at the locus to which metabolite maps are co‐annotated with the metabolite. We performed this analysis using two independent public databases KEGG (http://www.genome.jp/kegg) and HMDB (http://www.hmdb.ca) (Wishart *et al*, 2009). From the total of 240 associations, 3 associations (1%) were found to be validated by the public database annotation of biochemical pathways. The three validated associations included pyridoxate mapping to the chromosome 1 locus containing *aldehyde oxidase 1* (*Aox1*), glycerol 3‐phosphate mapping to chromosome 2 locus containing *glycerol‐3‐phosphate dehydrogenase 2* (*Gpd2)*, and N‐acetylglutamate mapping to chromosome 9 locus containing *aminoacylase 1* (*Acy1*).

**Figure 4:**
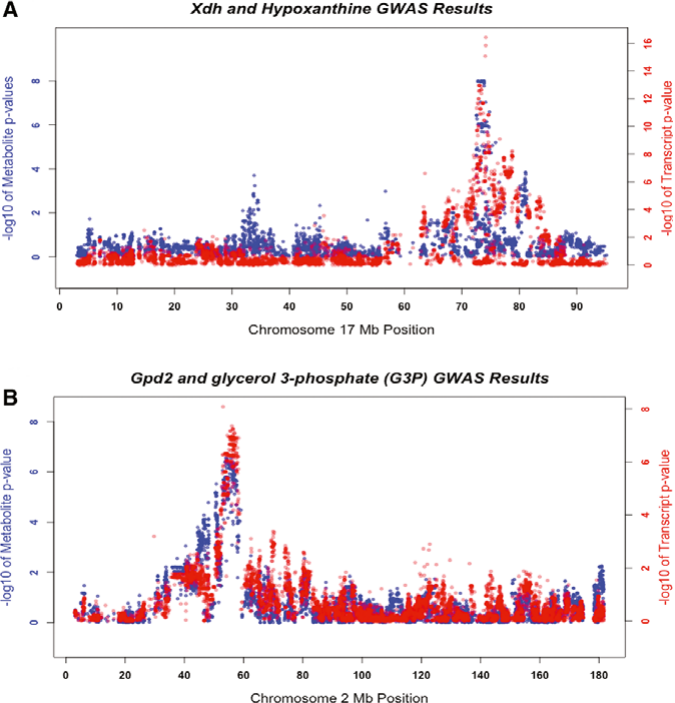
Validation of metabolite loci by the local eQTLs Co‐localization of *xanthine dehydrogenase *mRNA (red) with its product hypoxanthine (blue), at the chromosome 17 locus where the xanthine dehydrogenase gene, *Xdh1* resides.Co‐localization of *glycerol‐3‐phosphate dehydrogenase 2 *mRNA (red) with its substrate glycerol 3‐phosphate (blue), at the chromosome 2 locus where *glycerol‐3‐phosphate dehydrogenase 2* gene (*Gpd2*) resides.

In the second approach we searched in the expression data for any local eQTL that coincided with each of the 227 loci identified for the metabolites. As such, local eQTLs are useful in narrowing down the list of candidate genes at each locus. The local eQTLs utilized in this study were identified using the 5% FDR cutoff (*P*‐value = 1.7 × 10^−5^) and map within a megabase of the gene whose expression is affected. From the 227 loci identified, local eQTL analysis identified at least one candidate gene for 174 of the loci (~75% of the loci). We report these 174 loci along with the local eQTL candidate genes in Supplementary Table S4, and, below, we describe two specific examples in which the metabolite locus candidate gene was supported by the local eQTL data (Fig 4A and B). The first example is for the “hypoxanthine” metabolite. This metabolite, which belongs to the “Nucleotide” class of metabolites, can be produced in the cell either from xanthine or by degradation of purine. The former reaction is catalyzed by the enzyme xanthine oxidase (also known as xanthine dehydrogenase or Xdh), which is encoded by the *Xdh* gene located on chromosome 17. In HMDP mice, there is a genetic variation in this gene that affects the transcript levels of this gene, with a significant eQTL. It appears that this variation is also affecting the hypoxanthine pool in the liver of HMDP mice as hypoxanthine maps to the same locus as both the *Xdh* gene and the mRNA variation of *Xdh* (Fig 4A). The second example is for the locus on chromosome 2 where *glycerol‐3‐phosphate dehydrogenase 2* gene (*Gpd2)* resides. The protein product of this gene, which is localized to the mitochondrial inner membrane, functions in the glycerophospholipid metabolism pathway, and catalyzes the conversion of glycerol‐3‐phosphate (G3P) to dihydroxyacetone phosphate, using FAD as a cofactor. HMDP mice have a DNA variation which affects the transcription of this gene, as is evident by the local eQTL for *Gpd2*. In the metabolite GWAS results, G3P, the *Gpd2* substrate, maps to the exact location as *Gpd2* mRNA, suggesting that the same variation that affects *Gpd2* transcription also affects the G3P abundance in the liver of HMDP mice (Fig 4B).

### Candidate gene validation

Searching public databases coupled with transcriptome and metabolome mapping data resulted in a list of candidate genes regulating metabolites in liver. In order to evaluate the validity of our candidate gene identification approach, we performed biological validation of selected candidate genes by adenovirus overexpression. Since our aim was to demonstrate proof of principle rather than novel gene discovery, our selection of candidates was based on prior biochemical knowledge. The three candidate genes selected for this purpose were *Gpd2*,* Aox1*, and *Acy1*, and based on mapping locations they were predicted to affect glycerol‐3‐phosphate, N‐acetylglutamate, and pyridoxate metabolite levels in the liver, respectively. For each experiment, 4 C57BL/6J mice were injected with adenovirus expressing the candidate gene of interest and 6 days post injection the liver tissues were collected and metabolites profiled by the same methodology used for profiling metabolites of the HMDP strains (for details see Materials and Methods). An adenovirus expressing LacZ was used as a control.

**Figure 5:**
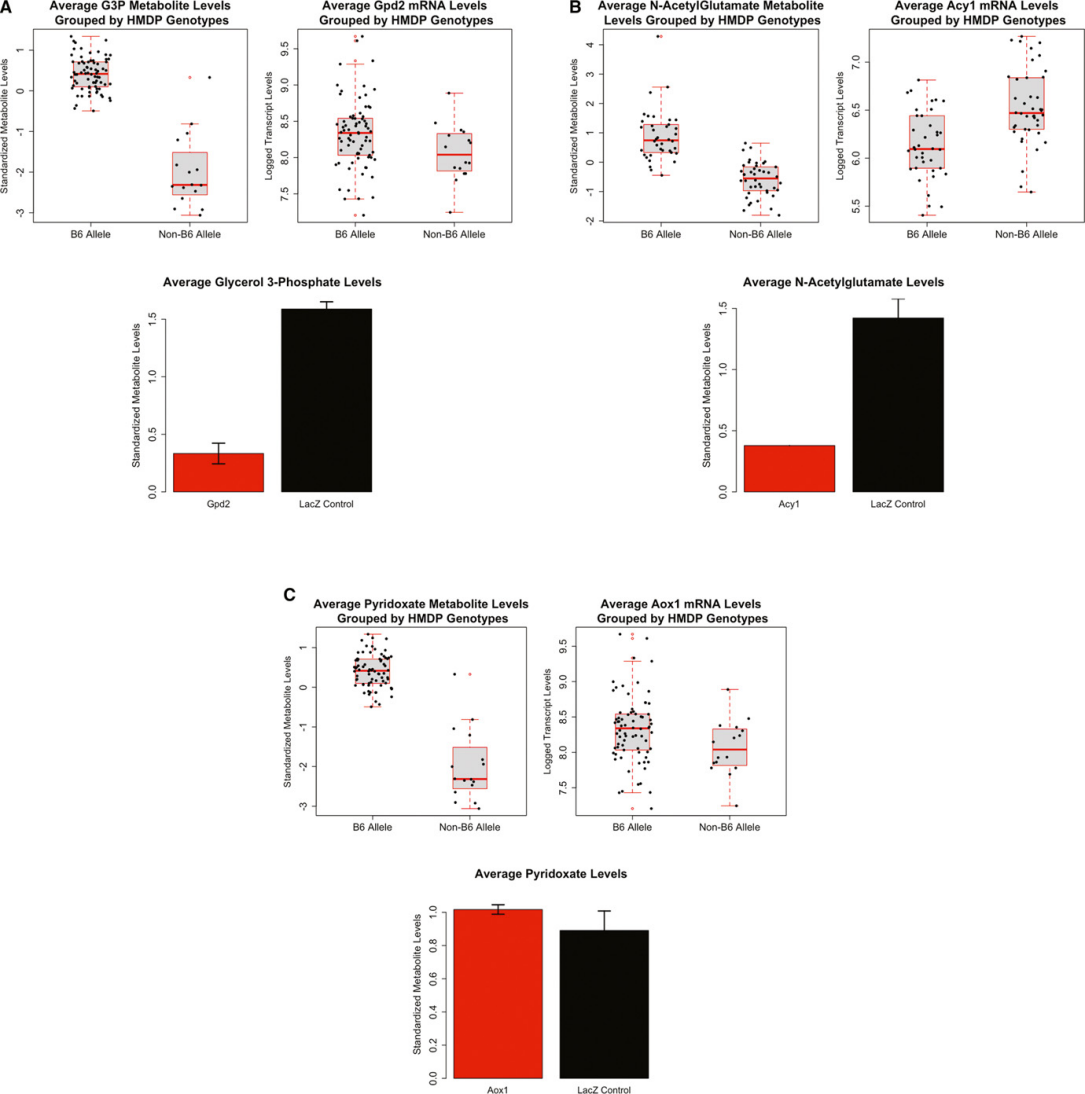
Biological validation of candidate genes Biological validation for the *Gpd2* gene and its effect on liver G3P levels. The GWAS results for the G3P metabolite levels and *Gpd2 *mRNA levels are shown on the top. On the bottom the results of adenovirus experiments in mice overexpressing *Gpd2* (red) and control mice (black) are shown. For each group four mice were used (*n* = 4).Biological validation for the *Acy1* gene and its effect on liver N‐acetylglutamate levels. The GWAS results for the N‐acetylglutamate metabolite levels and *Acy1 *mRNA levels are shown on the top. On the bottom the results of adenovirus experiments in mice overexpressing *Acy1* (red) and control mice (black) are shown. For each group four mice were used (*n* = 4).Biological validation for the *Aox1* and its effect on liver pyridoxate levels. The GWAS results for the pyridoxate metabolite levels and *Aox1 *mRNA levels are shown on the top. On the bottom the results of adenovirus experiments in mice overexpressing *Aox1* (red) and control mice (black) are shown. For each group four mice were used (*n* = 4).

As shown in Fig 5A, *Gpd2* overexpression by adenovirus in mice resulted in a significant reduction of glycerol‐3‐phosphate levels in liver compared to the control mice. These results were consistent with the GWAS data in which mice with the high‐expressor genotypes for *Gpd2* had significantly reduced G3P levels.

In the second experiment, adenoviral overexpression of *Acy1* gene in the liver of mice also resulted in changes predicted by the GWAS results. As shown in Fig 5B, GWAS data predicted that mice with the allele expressing higher levels of *Acy1* have significantly lower N‐acetylglutamate levels compared to mice carrying the variant that lowers *Acy1* levels. Consistent with this observation, adenoviral overexpression of *Acy1* in mice significantly lowered the N‐acetylglutamate levels in the liver, establishing the causal relationship between the *Acy1* gene and N‐acetylglutamate metabolite.

Lastly, GWAS data predicted that mice with higher *Aox1* transcript levels would have elevated pyridoxate levels. We observed slightly elevated levels of pyridoxate in mice overexpressing *Aox1* (Fig 5C) although these changes were not statistically significant. We note that 28 other local eQTL, including two other amylase gene family members, *Aox3* and *Aox4*, were present at the locus to which pyridoxate levels mapped and, thus, it is possible that the locus contains multiple genes influencing pyridoxate levels.

In summary, 2 of 3 validation experiments confirmed the effect of the candidate genes identified in the GWAS study, and the third was suggestive.

### Comparison of mouse and human loci

An immediate question arising from the results obtained in metabolite profiling of mouse liver data is whether the loci identified correspond to the loci regulating metabolite levels in human. Although no current human study is available in which liver metabolites have been measured, Illig *et al* recently published GWAS results for 163 blood serum metabolites in two large European populations (Illig *et al*, 2010) and subsequently Suhre *et al* published a meta‐analysis of these GWAS results and expanded the metabolite profiles to over 250 (Suhre *et al*, 2011). As the second report includes more metabolites we compared our results with theirs. In this meta‐analysis, authors reported 37 significant loci passing the genome‐wide significance. From these 37 loci, 17 loci regulated 17 separate single metabolite levels and 20 loci regulated the ratio of pair of metabolites. To investigate human‐mouse concordance in genetic regulation of metabolites, we first asked from the 37 significant loci in human how many replicated in our study. Of these, eleven metabolites were also measured in our study and from these eleven, five metabolites (succinylcarnitine, isobutyrylcarnitine, proline, carnitine, and isovalerylcarnitine) had at least one significant locus in the mouse GWAS results. When searched for the human syntenic loci in mouse for these five metabolites, succinylcarnitine was the only metabolite that was found to significantly map to the syntenic region in mouse (*P*‐value of 1.98e‐10) (Supplementary Table S5).”

Alternatively, we also looked at reproducibility of 119 mouse liver metabolites found to be significantly mapping to 240 loci in our study by looking at the GWAS results published by Suhre *et al* (2011) for single metabolites. From the 119 metabolites 54 metabolites were also measured in the human study. These common metabolites mapped to 115 distinct loci in mice. For each of these loci and their corresponding metabolites we asked if a human syntenic region harbors evidence of association. For the purpose of this comparison we relaxed the significance criteria in human data from genome‐wide cutoff to any GWAS *P*‐value result ≤ 4.3 × 10^−4^ as set by Bonferoni correction for the 115 loci (0.05 divided by 115). From the 115 total loci, we found evidence of GWAS association for 39% of these loci (45 out of 115) (Supplementary Table S6). Out of the 45 loci, 40 loci were found in only one of the two populations (either TwinUK or Kora) and for 5 loci we found significant evidence in both populations. This included a highly significant locus on chromosome 9 in mouse for succinylcarnitine. In humans this metabolite mapped to the syntenic region on chromosome 15 (61.2 Mb) with the GWAS *P*‐values of 6.8e‐21and 1.5e‐07 in the KORA and TwinUK populations, respectively. The variation at this locus in mouse explained 32% of total succinylcarnitine variation in the HMDP. It is worth noting that, from the four mouse metabolites mapping to the loci that explained more than 50% of the variations, one locus (the mouse chromosome 1 locus regulating pyridoxate) was also found in the syntenic region in the KORA population, with the *P*‐value of 1.9e‐04. We were unable to examine the other three loci as the metabolites mapping to these loci were not measured in human.

It has been suggested that mapping metabolite ratios instead of single metabolites may improve the mapping results in GWAS. To investigate this, we mapped the metabolite ratios which had at least one genome‐wide significance in the human blood serum metabolite meta‐analysis (Suhre *et al*, 2011) and asked whether the mapping results are significantly improved compared to mapping single metabolite levels. As shown in Supplementary Fig S3, we found no marked improvement in GWAS results when ratios of two related metabolites were mapped compared to single metabolite levels. The only instance in which mapping ratios resulted in significant result was for the Docosahexaenoate/Eicosapentaenoate ratio which mapped to the locus on Chromosome 14 (Supplementary Fig S3). In addition, none of the highly significant human loci published for metabolite ratios replicated for their corresponding metabolite ratios in mice (Supplementary Table S5).

### Correlations between metabolites, transcripts, and clinical traits

In an effort to understand the relationship between gene expression, metabolite levels, and clinical traits using a “systems genetics” approach (Civelek & Lusis, 2014), we integrated metabolite levels with gene expression microarray data and clinical trait data that were available for the HMDP. We studied the relationship between transcript and metabolite levels by calculating pair‐wise correlations between mRNA levels and the metabolites abundance. At the 5% FDR (*P*‐value of 1.28e‐05), we found a total of 1,497 significant metabolite‐mRNA correlations of which 721 (48%) were positive and 776 (52%) were negative. The 1,497 significant correlations included 969 probesets on the gene expression microarray and 145 metabolites. To investigate the significance of integrating metabolite and transcript data, we also correlated each of these molecular phenotypes with 58 metabolic syndrome related clinical traits (Supplementary Table S7). The clinical traits utilized in this study are part of our ongoing effort to collect HMDB phenotypes with the focus on traits related to cardiovascular and metabolic disorders. At a cutoff of 5% FDR (*P*‐value = 1e‐04), we found 36 significant correlations. These included both known (such as lactate‐plasma glucose, fructose‐plasma glucose, 7‐beta‐hydroxycholesterol‐plasma unesterified cholesterols, and betaine‐fat pad mass) and novel metabolite‐trait relations (such as glycerate‐bone density, and ascorbate‐plasma total cholesterol levels). Two‐dimensional hierarchical clustering of transcripts against metabolites and clinical traits revealed discrete clusters of mRNA‐metabolite‐trait relationships (Supplementary Fig S4).

## Discussion

In the past decade, major efforts have been focused on developing efficient methods to extract and quantify metabolite profiles from various tissues (Wang *et al*, 2010). Several recent reports have investigated the utility of metabolome in discovering genetic variations underlying human disease, biomarkers for cancer, and biomarkers for pharmacological responses (Gieger *et al*, 2008; Bain *et al*, 2009; Illig *et al*, 2010; Suhre *et al*, 2011; Wang *et al*, 2011). One limitation of the human studies is that these studies mainly measured metabolites in blood serum. Here, we investigated the relationships of a large set of metabolites encompassing major metabolic processes in the mouse liver, a central tissue for metabolism. Overall we were able to map approximately 40% of the metabolites to at least one locus in the genome. Our study shows that the majority of loci regulating these metabolites accounted for 20–40% of total metabolite variation and a few (4 loci) accounted for more than 50% variation. This contrasts to loci regulating physiological/clinical traits which generally explain 10% or less of the phenotypes. In principal, genetic effect sizes are expected to be larger for molecular, or “intermediate”, phenotypes, such as metabolites or transcript levels, as compared to clinical phenotypes, and to be larger in experimental organisms, where environmental factors can be reduced as compared to human studies. The latter may also explain why we found many more loci regulating metabolite levels in mice compared to the recent human studies (240 significant loci found in our study versus 37 in the human report). Moreover, we found evidence for similarity in genetic regulation of metabolites in mouse and human for 39% of the loci found to be significant in mice. However, 61% of loci that were found to be significant in our study did not replicate in the human published studies. The observed dissimilarities between the mouse data and the human can be partly explained by interspecies differences between mouse and human, and partly by the study design differences where serum metabolites were mapped in human whereas we report on loci regulating liver metabolites.

The study described in this manuscript has several limitations. First, the list of prioritized candidate genes was mainly derived from the overlapping cis‐eQTL data. As this list would only support genetic variations affecting transcript levels, candidate genes regulated at the protein level will not be included in this list. To compensate for that, we queried the prior knowledge from publicly available databases, but this approach only added minimally to prioritization of candidate genes reflecting our incomplete state of knowledge about the genetic factors regulating metabolite levels and supporting the limited utility of the curated databases to find potential candidate genes. An alternative approach to find candidate genes would be to take a network based approach which would allow for identification of genes driving network modules comprised of metabolites and other nodes (i.e. transcripts or clinical traits). Initial studies taking this integrative approach have proved useful in inferring the drivers of network module by combining genetic variation with metabolite and transcriptome data (Inouye *et al*, 2010).

Another limitation of our study was the use of limited number of replicates in measuring metabolites for each inbred strain. The presence of biological replicates among the population of genetically distinct inbred mice allows one to accurately assess the variation in metabolites due to genetic factors. However due to practical limitations we were able to only include five biological replicates for only one of the strains in our study. Also, since we used a *P*‐value threshold based on FDR, some of our findings (12/119 loci) are likely to correspond to false positives. Another limitation of the current study includes the absence of environmental perturbation. We have shown that in mice physiological response to environmental perturbations (such as diet) may trigger response differently depending on the genetic make up of the organism (Parks *et al*, 2013). In addition, evidence suggests that in genetically diverse populations diet might be a major factor accounting for metabolite variation (Holmes *et al*, 2008). Moreover, metabolites relating to mitochondrial energy metabolism were found to differentiate gender and age (Slupsky *et al*, 2007). In our study we only focused on the effect of natural variation on liver metabolites in the absence of both sex differences and environmental perturbations. For future studies, however, the HMDP population offers an ideal resource to elucidate the extent of the interaction between environment and genes to regulate metabolite levels. This resource is also ideal for examining the effect of age and sex on metabolite profile and our future studies are aimed at addressing these questions. Lastly, it has been shown that there is a great within‐individual longitudinal variation compared to population variation in the human urine metabolite levels (Nicholson *et al*, 2011). The design of our study, however, prevented us from examining such variation within each mouse as our study was performed on the liver which could not be resampled over time.

In our study, we found significant intra‐ and inter‐class correlations between metabolites, reflecting shared biochemical pathways or regulatory interactions. This was especially true for the metabolites classified as Lipids, Amino Acids, and Carbohydrates. The presence of significant correlations between metabolites categorized in two separate classes presumably reflects either multiple roles of metabolites or interactions between metabolic pathways.

Our study highlights the value of integrating data from various biological scales, such as metabolome, transcriptome, phenome, and genome. The integration of transcriptome data and the corresponding eQTL profile assisted us in reducing the number of candidate genes residing under each metabolite locus. The validity of this approach was highlighted by the biological validation of two of the candidate genes identified. In each validation experiment the results were in complete concordance with what we had found in the GWAS results, both supporting the validity of our approach and providing a resource that should aid in identifying the genetic factors affecting metabolite variations. The results of validation experiments are also consistent with the biochemical function of the tested candidate genes. *Gpd2* is an enzyme that belongs to the glycerol phosphate shuttle and catalyzes the conversion of glycerol‐3‐phosphate (G3P) to dihydroxyacetone phosphate. Therefore, it is expected that the mice with high expression of this enzyme will have lesser amount of the G3P substrate. This hypothesis was also supported by the GWAS results and subsequently shown to be true by our validation experiment. Interestingly, St‐Pierre *et al* (2001) has published evidence for association between variation in *Gpd2* and free fatty acid concentration in French Canadians. Accordingly we examined the previously published free fatty acid GWAS results in HMDP and found a suggestive LOD score for this trait on chromosome 2 where G3P and *Gpd2* map and where the *Gpd2* gene is physically located (data not shown).

Similarly, our biological validation of *Acy1* supported the hypothesis generated by the GWAS results. The GWAS data predicted that this enzyme was affecting the levels of N‐acetylglutamate in the liver. According to the KEGG pathway database, *Acy1* is an intermediate enzyme in the biochemical pathway that converts glutamine to ornithine, with the latter being subsequently utilized in the Urea Cycle. Therefore, as expected, mice that express higher levels of *Acy1* will be expected to have lower levels of N‐acetylglutamate. Our validation corroborated this expectation and provided a causal link between variation in *Acy1*, an intermediate enzyme in the conversion of glutamine to ornithine, and N‐acetylglutamate. Interestingly, the ornithine levels neither mapped to the *Acy1* locus nor were they changed in the mice injected with adenovirus carrying the *Acy1* construct. This perhaps is due to the complex regulation of ornithine levels in liver, as this metabolite is being used in Urea Cycle and can be produced by means other than conversion of glutamine.

In our third validation experiment, *Aox1* overexpression, was predicted to affect pyridoxate levels. Although we did observe a change in pyridoxate levels consistent with the GWAS data, the difference did not reach statistical significance. In the GWAS analysis, pyridoxate levels mapped to chromosome 1 at 58 Mb locus with highly significant *P*‐value of 1.66e‐28. A closer examination of this locus revealed that in addition to *Aox1*, there are several other candidate genes, including two other members of the amylase gene family with local eQTLs in this region.

Overall, our study complements other recent reports in establishing the influence of genetic variation on metabolites and provides support for the utility of metabolome integration with transcriptome to elucidate novel relationships across various biological scales. Investigation of metabolite levels will clearly be important in establishing the flow of information that underlies common diseases. In particular, human GWAS studies have identified thousands of loci contributing to common diseases, and a major challenge at present is to understand the pathways that are perturbed. A “systems genetics” approach as described here should complement more traditional “one‐gene‐at‐a‐time” approaches (Civelek & Lusis, 2014).

## Materials and Methods

### Clinical trait measurements

Animals and Clinical Phenotypes: Male mice from the HMDP panel, approximately 6‐10 weeks of age, were purchased from Jackson Laboratory and were fed Purina Chow (Ralston‐Purina Co., St. Louise, MO) containing 4% fat until sacrifice. All mice were maintained on a 12 h light/dark cycle. At 16 weeks of age, whole body fat, fluids and lean tissue mass of mice were determined using a Bruker Optics Mini spec nuclear magnetic resonance (NMR) analyzer (The Woodlands, TX, USA) according to the manufacturer's recommendations. We also calculated the total mass of the mice, sum of lean mass, free fluid, fat mass, body fat percentage, and fat mass/total mass. Following a 16‐h fast, mice were weighed and then bled retro‐orbitally under isoflurane anaesthesia. Complete blood counts were performed using a Heska CDC‐Diff analyzer (Heska Corp, Loveland, CO, USA). Plasma lipids were determined as previously described (Mehrabian *et al*, 1993). Glucose levels were determined using commercially available kits from Sigma (St. Louis, MO, USA). Insulin levels were measured using commercial ELISA kits (ALPCO Diagnostics). All measurements were performed in triplicate according to the manufacturer's instructions. Mice were euthanized by cervical dislocation and the mass of individual tissues and fat depots (heart, kidney, retroperitoneal fat pad, epididymal fat pad, subcutaneous fat pad, and omental fat pad) were determined by dissecting and weighing each tissue/pad separately after the mice were euthanized. Following this, liver tissues were dissected out, flash frozen in liquid nitrogen, and kept at −70°C until further processing. Bone Mineral Density (BMD) was determined as previously described (Farber *et al*, 2011). Plasma Apolipoprotein levels of *ApoA1*,* ApoA2, ApoA4, ApoB, ApoC2, ApoC3, ApoD, ApoE, ApoM, Clusterin, LCAT, LpPLA2, PON1*, and *SAA1* were determined by the Protein Analysis Research Center (PARC) at Indiana University School of Medicine. Briefly, 100 μl aliquots of mouse plasma were used to determine apolipoprotein abundance using an MRM‐based targeted proteomic approach. After constant amount of human apolipoprotein was spiked into each sample, all apolipoproteins were enriched and digested with trypsin using AB/SCIEX 4000 QTRAP and Dionex U3000 HPLC system. A microspray source was used for all the MS analyses. Source temperature was set at 450°C, and source voltage was set at 5,500 V. Collision Energy (CE) and Declustering Potential (DP) for each transition were automatically calculated by the Skyline algorithm. Transitions for each peptide were averaged for each sample on the log2 Area‐Under‐the‐Curve (AUC) scale to get a single number for peptide level for each sample. A relative amount of protein was calculated against commercially available pooled healthy mouse plasma. The analysis was carried out using JMP. *LCAT* activity was measured using a kit according the manufacturers' specifications (Roar Biomedical, Inc., New York, NY). *PON* activity was determined as (Shih *et al*, 1998). *Phospholipid Transfer Protein* (*PLTP*) was determined using a commercially available kit (# P7700 from Roar Biomedical, Inc., New York).

All animals were handled in strict accordance with good animal practice as defined by the relevant national and/or local animal welfare bodies, and all animal experiments and work were carried out with UCLA IACUC approval.

### Metabolic profiling

A total of 104 strains were used for metabolite profiling. All mice were fasted overnight and liver samples were harvested between 10 AM and 12 PM the next day. Liver samples were then shipped to Metabolon and the profiling was completed in 6 days. For the majority of strains used in this study we only measured metabolites in the liver of one mouse per strain. For 20 strains, however, we profiled more than one mouse per strain. The strains with more than one mouse profiled includes: AJ, AXB19b, AXB24, B6, BALB/c, BXA16, BXD19, BXH4, C3H, CXB1, CXB11, CXB12, CXB13, CXB2, CXB3, CXB4, CXB6, CXB7, CXB8, CXB9, and DBA (all except B6 had 2 mice per strain). For the strains with more than one mouse per strain, we used the mean of the liver metabolite across the mice to represent the metabolite value. For the B6 strain, in particular, we included five mice to estimate the intrastrain (nongenetic) variation.

The non‐targeted metabolic profiling instrumentation employed for this analysis combined three independent platforms: ultrahigh performance liquid chromatography/tandem mass spectrometry (UHPLC/MS/MS2) optimized for basic species, UHPLC/MS/MS2 optimized for acidic species, and gas chromatography/mass spectrometry (GC/MS). Samples were processed essentially as described previously (Evans *et al*, 2009; Ohta *et al*, 2009). For each sample, 100 μl was used for analyses. Using an automated liquid handler (Hamilton LabStar, Salt Lake City, UT), protein was precipitated from the tissue water homogenate with methanol that contained four standards to report on extraction efficiency. The resulting supernatant was split into equal aliquots for analysis on the three platforms. Aliquots, dried under nitrogen and vacuum‐desiccated, were subsequently either reconstituted in 50 μl 0.1% formic acid in water (acidic conditions) or in 50 μl 6.5 mM ammonium bicarbonate in water, pH 8 (basic conditions) for the two UHPLC/MS/MS2 analyses or derivatized to a final volume of 50 μl for GC/MS analysis using equal parts bistrimethyl‐silyl‐trifluoroacetamide and solvent mixture acetonitrile: dichloromethane: cyclohexane (5:4:1) with 5% triethylamine at 60°C for 1 h. In addition, three types of controls were analyzed in concert with the experimental samples: aliquots of a pooled sample derived from a portion of all experimental samples served as technical replicates throughout the data set, extracted water samples served as process blanks, and a cocktail of standards spiked into every analyzed sample allowed instrument performance monitoring. Experimental samples and controls were randomized across platform run days.

For UHLC/MS/MS2 analysis, aliquots were separated using a Waters Acquity UPLC (Waters, Millford, MA) and analyzed using an LTQ mass spectrometer (Thermo Fisher Scientific, Inc., Waltham, MA) which consisted of an electrospray ionization (ESI) source and linear ion‐trap (LIT) mass analyzer. The MS instrument scanned 99–1,000 m/z and alternated between MS and MS2 scans using dynamic exclusion with approximately six scans per second. Derivatized samples for GC/MS were separated on a 5% phenyldimethyl silicone column with helium as the carrier gas and a temperature ramp from 60 to 340°C and then analyzed on a Thermo‐Finnigan Trace DSQ MS (Thermo Fisher Scientific, Inc.) operated at unit mass resolving power with electron impact ionization and a 50–750 atomic mass unit scan range.

In order to account for run‐to‐run variation across instruments and day‐to‐day variation, well characterized internal standards were spiked into each sample. The compounds used as internal standards are chosen so as not to interfere with the measurement of the endogenous compounds in the sample. In addition, a cocktail of homogeneous pool containing a small amount of all study samples served as technical replicates throughout each run. This cocktail was sent across the GC/MS, and LC/MS/MS2 instruments 5–6 times per run day as a mechanism to monitor variation within runs and between runs. The use of technical replicates revealed that the instrument variability was 5% and overall variability (defined as within and between run variability) was 9%. To account for such variability and to standardize data across runs, data were corrected by registering the median of each run‐day to one followed by normalizing each data point proportionately.

Metabolites were identified by automated comparison of the ion features in the experimental samples to a reference library of chemical standard entries that included retention time, molecular weight (m/z), preferred adducts, and in‐source fragments as well as associated MS spectra, and were curated by visual inspection for quality control using software developed at Metabolon (Dehaven *et al*, 2010).

This methodology enabled us to quantify a comprehensive set of 342 metabolites present in mouse liver tissues across the HMDP panel. In order to minimize false positive associations in our subsequent genome‐wide association study, we filtered out the metabolites that exhibited significant numbers of missing values (> 20%) in the samples profiled. As a result, 59 of the metabolites were filtered out and the remaining 283 metabolites were selected for further analysis.

### RNA isolation and expression profiling

At 16 weeks of age, the liver tissues of the mice were dissected and flash frozen in liquid nitrogen, and kept at −70°C until further processing. For RNA profiling, the RNA from three mice per strain were hybridized to Affymetrix Mouse Genome HT_MG‐430A arrays. Frozen liver samples were weighed and homogenized in Qiazol according to the manufacturer's protocol. Following homogenization, RNA extraction was performed using Qiagen's RNeasy kit (cat# 74104). 92 strains of mice had three biological replicates, five strains had two biological replicates and two strains with one biological replicate each. All RNA samples were cleaned using a Biosprint96 (Qiagen, Valencia, CA) with RNA cleanup beads (Agencourt Bioscience, Beverly, MA) following manufacturer's protocol with adaptations for use with the Biosprint. The quality of the total RNA from the those samples were monitored by the Agilent 2100 Bioanalyzer (Agilent Technologies, Palo Alto, CA) and RNA quantity was measured with a NanoDrop (NanoDrop Technologies, Inc., Wilmington, DE) following the manufacturer's instructions. All samples were arrayed into three 96‐well microtiter plates following a randomized design format that places samples from the same strain on different plates to better estimate variance across testing strains. All target labeling reagents were purchased from Affymetrix (Santa Clara, CA). Double‐stranded cDNAs were synthesized from 1μg total RNA through reverse transcription with an oligo dT primer containing the T7 RNA polymerase promoter and double strand conversion using the cDNA Synthesis System. Biotin‐labeled cRNA was generated from the cDNA and used to probe Affymetrix Mouse Genome HT_MG‐430A arrays. The HT_MG‐430A Array plate consists of 96 single MG‐430A arrays arranged into standard SBS 96‐well plate format. All cDNA and cRNA target preparation steps were processed on a Caliper GeneChip Array Station from Affymetrix. Array hybridization, washing and scanning were performed according to the manufacturer's recommendations. Scanned images were subjected to visual inspection and a chip quality report was generated by the Affymetrix's GeneChipOperating System (GCOS) and Expression console (Affymetrix). Two of 288 chips were excluded due to low QC scores. The image data was processed using the Affymetrix GCOS algorithm utilizing quantile normalization or the Robust Multiarray method (RMA) to determine the specific hybridizing signal for each gene. Expression data can be obtained from Geo database (GSE16780). To avoid the effect of SNP on hybridization, we matched the location of approximately 14 million SNPs from dbSNP database (NCBI) to the location of the individual probes on the genome. If the location of the probe had a matching SNP within it, we flagged the probe and excluded it from the cdf file prior to RMA normalization. If a probeset contained SNP in 8 or more 25‐mer probes, we excluded the probeset from the analysis. The cleaned datasets were then background corrected and normalized using the affy package (from bioconductor) using rma, pmonly, and median‐polish normalization methods.

### Genotyping and genome‐wide association mapping

Inbred strains were previously genotyped by the Broad Institute (http://www.broadinstitute.org/mouse/hapmap), and they were combined with the genotypes from Wellcome Trust Center for Human Genetics (WTCHG). Genotypes of RI strains at the Broad SNPs were inferred from WTCHG genotypes by interpolating alleles at polymorphic SNPs among parental strains, calling ambiguous genotypes missing. Of the 140,000 SNPs available, 107,145 were informative with an allele frequency > 5%. These SNPs were used for metabolite, transcript, and clinical trait genome‐wide association analysis.

We applied the following linear mixed model to account for the population structure and genetic relatedness among strains in the genome‐wide association mapping (Kang *et al*, 2008a,b): y=μ+xβ+u+e

In the formula, μ represents mean, x represents SNP effect, u represents random effects due to genetic relatedness with Var(u) = σg2K and Var(e) = σe2, where K represents IBS (identity‐by‐state) matrix across all genotypes in the HMDP panel. A restricted maximum likelihood (REML) estimate of σg2 and σe2 are computed using EMMA, and the association mapping is performed based on the estimated variance component with a standard *F*‐test to test β ≠ 0. We applied Efficient Mixed Model Association (EMMA) as an R implementation of a linear mixed model. The percent of variance explained for each molecular phenotype was calculated using the SNP effect calculated from EMMA by defining it as: 1 − [(variance of residuals)/(variance of original phenotypes)]. It should be noted that since EMMA is orders of magnitude faster than other implementations commonly used, we were able to perform statistical analyses for all pairs of transcripts/metabolites and genome wide markers in a few hours using a cluster of 50 processors.

The “peak SNP” reported for each metabolite was identified by first finding all the SNPs that exceeded the genome‐wide cutoff for that particular metabolite. Next, SNPs were ordered by the strength of association, and among the ranked SNPs the SNP *R*‐squares were calculated. Among the SNPs that had *R*‐square > 0.5, only the SNP that showed the strongest association was kept and reported as the “peak SNP” in the manuscript. To look for candidate genes for each metabolites, we took each metabolite's “peak SNP” at each significant locus and searched for the presence of local eQTL in the interval spanning from 1 Mb proximal to 1 Mb distal to the metabolite peak SNP. eQTL were defined as “local” if the peak association SNP position for the transcript was within a 4 Mb interval, flanking 2 Mb on either side of the transcription start and end of the gene under regulation.

Genome‐wide cutoff: Genome‐wide cutoffs were calculated as the false discovery rates using the “*q*‐value” package for FDR calculation in the R statistical software (Storey & Tibshirani, 2003). FDR calculation was carried out separately for the metabolites and transcript dataset. For transcript data, due to the computational complexity associated with evaluating *q*‐values for over 400 million *P*‐values, we computed the FDRs by taking the average FDR for 100 samples each containing 5 million randomly selected *P*‐values from the original calculated *P*‐values. The FDR threshold is similar to that calculated in the HMDP population using simulation or a Bonferroni correction for SNPs examined(Bennett *et al*, 2010). The fact that it is larger than the usual human GWAS threshold reflect the fact that the linkage disequilibrium blocks in the HMDP are larger than those in human populations(Bennett *et al*, 2010; Parks *et al*, 2013).

### Adenovirus generation, purification and infection *in vivo*

Recombinant adenovirus was generated using the AdEasy system as previously described (Bennett *et al*, 2013). Briefly, linearized shuttle vector containing full‐length mouse cDNA for each of the three candidate genes (*Gpd2*,* Acy1*, and *Aox1*) was transformed into *E. coli* BJ5183AD cells containing the adenoviral backbone plasmid pAdEasy‐1 for homologous recombination. Positive recombinants were linearized and transfected into HEK293AD cells for virus packaging and propagation. Adenoviruses expressing the candidate gene were purified by CsCl banding and stored at −80°C until use. For adenoviral infection, 10‐week‐old male C57BL/6 mice were injected with adenoviral construct (~2 × 10^9^ PFUs diluted in 0.2 ml saline) through the tail vein. After overnight fasting, mice were sacrificed 6 days post injection, the liver tissue was extracted, the expression of each candidate gene was assessed by RT‐PCR, and the metabolites were profiled by Metabolon, Inc (Durham, NC). For each candidate gene, we utilized four mice (*n* = 4). The control group consisted of mice injected with adenoviral construct expressing the LacZ gene.

### Other statistical methods/Software

The *z*‐scores for each metabolite in each mouse was calculated using standard formula: *z*‐score = (relative metabolite level in the sample – mean of metabolite level in the samples)/standard deviation of metabolite levels in the samples. The calculated *z*‐scores were used both in genome‐wide association and in correlation analysis.

Public databases utilized to pair metabolites with genes were Kyoto Encyclopedia of Genes and Genomes (KEGG) at http://www.genome.jp/kegg and Human Metabolome Database (HMDB) at www.hmdb.ca (Wishart *et al*, 2009). For KEGG, we searched the pathways specific to mouse. HMDB, however, is a freely available electronic database containing detailed information about small molecule metabolites found in the human body. To translate the information to mouse, we downloaded all the relationships documented between small molecules and genes in HMDB version 2.5 and converted all the human genes to mouse orthologous genes. The resulting mouse gene‐small molecule file was then used throughout the paper.

All statistical analyses, database annotation, and data visualizations were carried out using the R (version 2.13.1) statistical software (available at http://cran.r-project.org/) and Python 3.0 (http://python.org/). All the correlation coefficients and corresponding *P*‐values reported in the paper are calculated using the bicor function in the WGCNA R package (Langfelder & Horvath, 2008) The main advantage of using bicor, which performs biweight midcorrelation calculation, over Pearson's correlation is based the robustness of the correlation coefficient measurement to the presence of outliers in the data.

### Data accessibility

All the genome‐wide significant mapping results for metabolites along with the candidate genes regulating each metabolite are provided in Supplementary Tables and deposited in our website at http://systems.genetics.ucla.edu. In addition, the liver metabolite data from this publication have been submitted to the Mouse Phenome Database at http://phenome.jax.org and assigned the identifiers HMDPpheno6, HMDPpheno7, HMDPpheno8, HMDPpheno9, and HMDPpheno10. Liver mouse transcript data are deposited to the GEO at http://www.ncbi.nlm.nih.gov/geo/ and assigned the identifier GSE16780.

## Author contributions

BJB, AG, and AJL generated and analyzed the metabolite data. LO, BJB, AG, CP generated and analyzed the genome‐wide association data. TK, WY, PK, AH, RH, LO, AG, BJB, AJL generated and analyzed gene expression data. DS and NC generated the adenovirus and performed *in vivo* validation. CP generated the UCLA website for data access and assisted in data analysis. AG analyzed the human‐mouse comparison data. AG, BJB, and AJL wrote the manuscript.

## Conflict of interest

The authors declare that they have no conflict of interest.

## Supplementary Material

Supplementary Figure S1

Supplementary Figure S2

Supplementary Figure S3

Supplementary Figure S4

Supplementary Table S1

Supplementary Table S2

Supplementary Table S3

Supplementary Table S4

Supplementary Table S5

Supplementary Table S6

Supplementary Table S7

Review Process File
