# Supplementary material for: Genetic regulation of mouse liver metabolite levels
Source: Mol Syst Biol. 2014 May 23;10(5):730. doi: 10.15252/msb.20135004 (PMC4188043; doi:10.15252/msb.20135004)

Figure S1. Composition of the 283 metabolites measured in HMDP mouse liver. This pie chart shows the number of metabolites measured for each of the eight major classes (and the percentage of total).

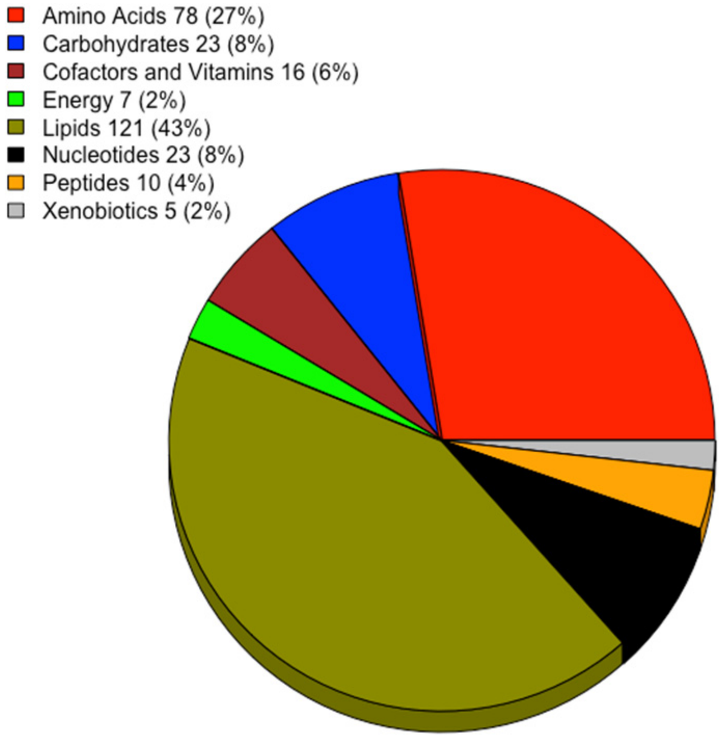

Supplement: Supplementary file 1 — Supplementary Figure S1 [file MSB-10-5-730-s1.pdf]
