# Supplementary material for: Genetic regulation of mouse liver metabolite levels
Source: Mol Syst Biol. 2014 May 23;10(5):730. doi: 10.15252/msb.20135004 (PMC4188043; doi:10.15252/msb.20135004)

Figure S2. Strength of Association for various classes of metabolites. This figure shows the strength of association of p-values for significant loci where metabolites are grouped together based on the class of metabolites they represent. The color key indicates the class of metabolite.

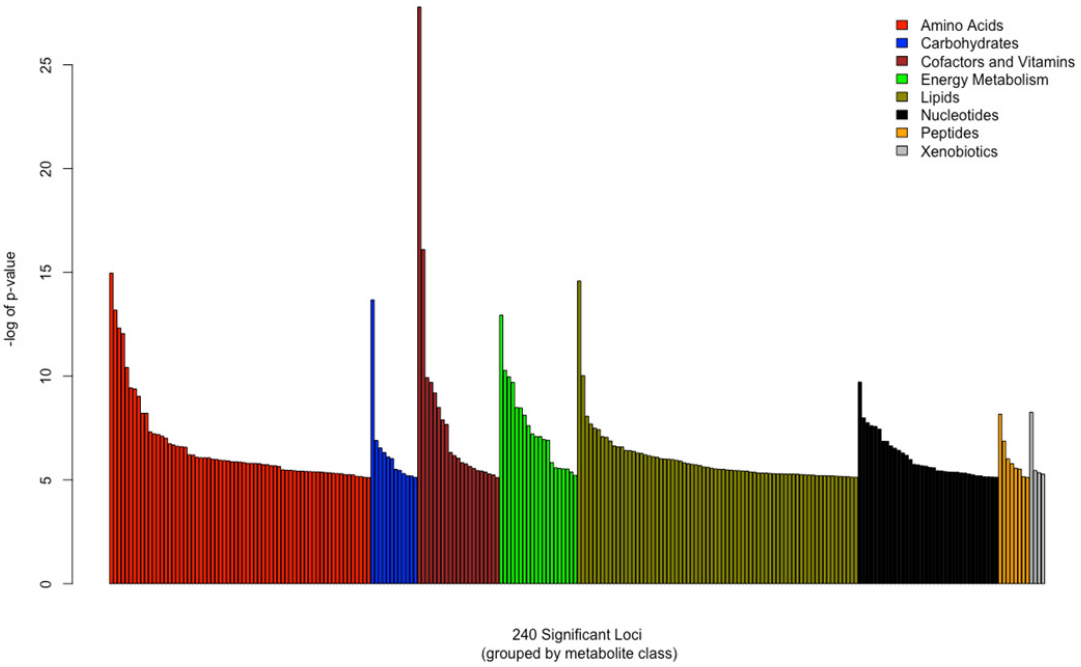

Supplement: Supplementary file 2 — Supplementary Figure S2 [file MSB-10-5-730-s2.pdf]
