# Supplementary material for: Genetic regulation of mouse liver metabolite levels
Source: Mol Syst Biol. 2014 May 23;10(5):730. doi: 10.15252/msb.20135004 (PMC4188043; doi:10.15252/msb.20135004)
Supplement: Supplementary file 3 — Supplementary Figure S3 [file MSB-10-5-730-s3.pdf]

Figure S3. Comparison of mapping results between mapping individual metabolites vs mapping metabolite ratios for pair of metabolites. In each panel (A-I) the top figure is the genomewide mapping result for the metabolite ratio of a pair of metabolites and the middle and the bottom figures are genomewide mapping results for each individual metabolite. The name of each metabolite is given on the top of each figure. In each figure the dashed line across represents the genomewide significance cutoff.

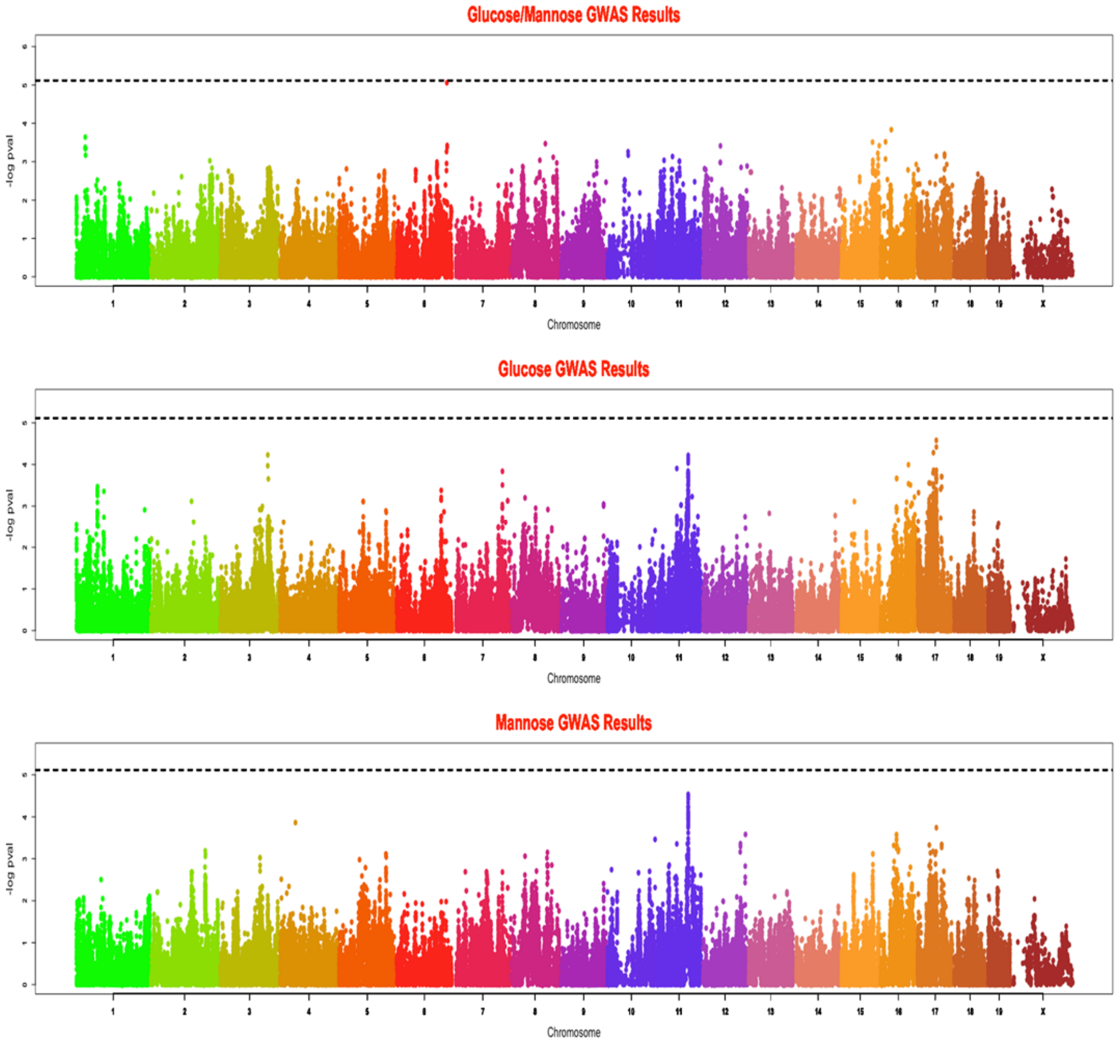

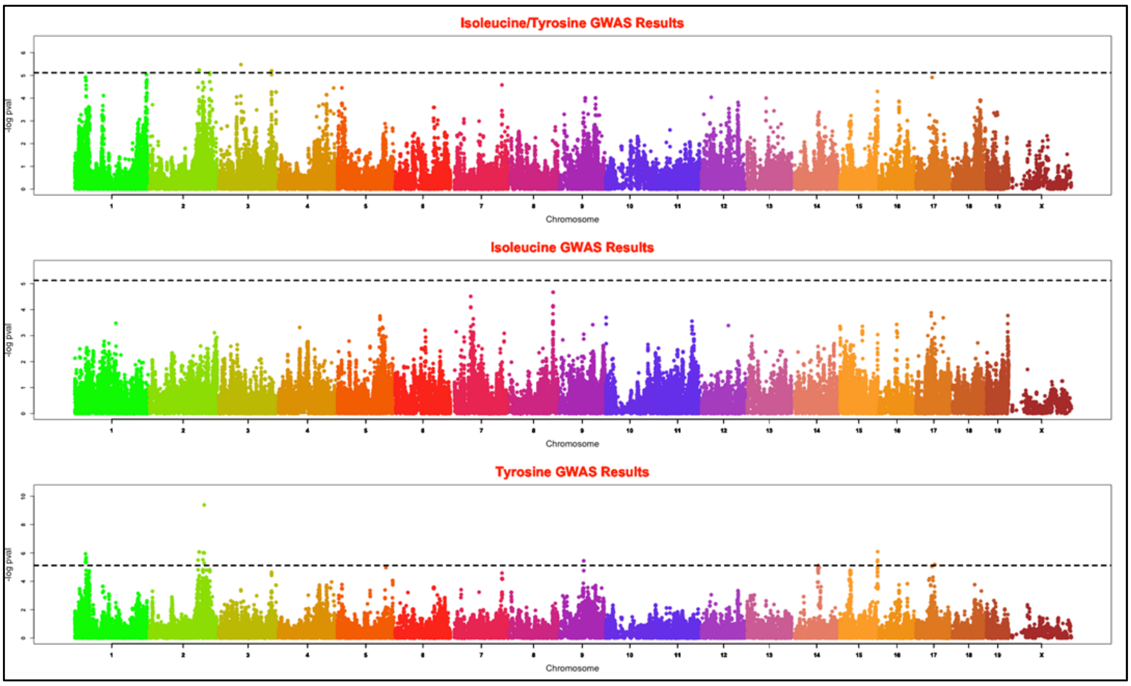
Isoleucine/Tyrosine GWAS Results
-log pval
Chromosome
Isoleucine GWAS Results
-log pval
Chromosome
Tyrosine GWAS Results
-log pval
Chromosome

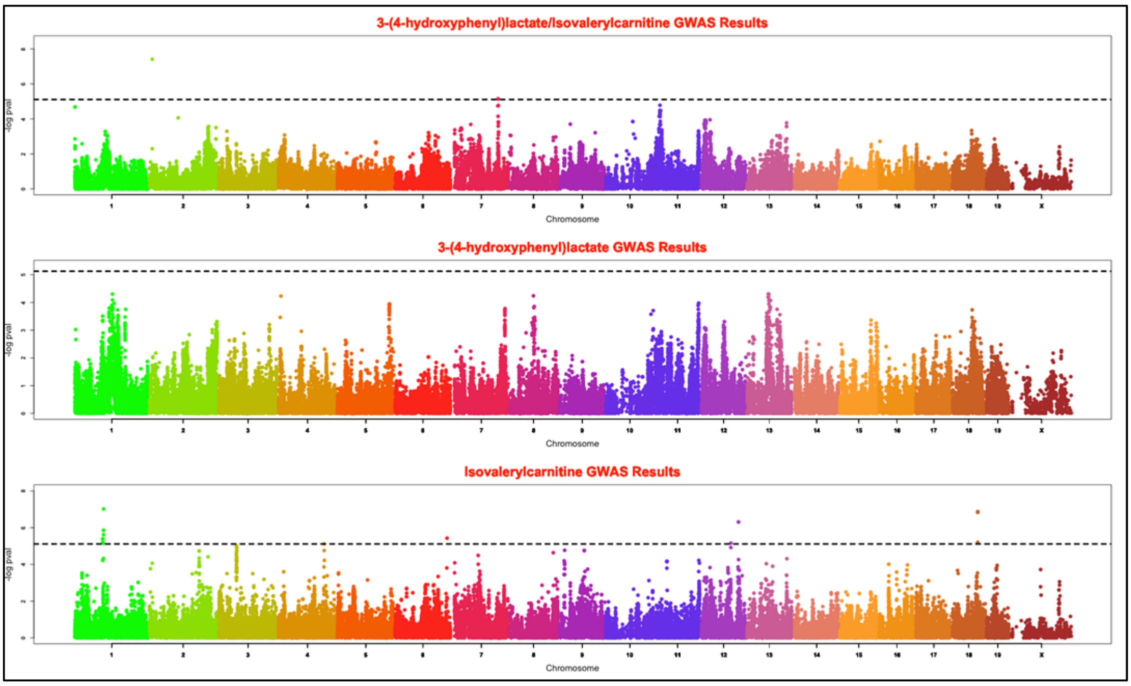
3-(4-hydroxyphenyl)lactate/Isovalerylcarnitine GWAS Results
-log pval
Chromosome
3-(4-hydroxyphenyl)lactate GWAS Results
-log pval
Chromosome
Isovalerylcarnitine GWAS Results
-log pval
Chromosome

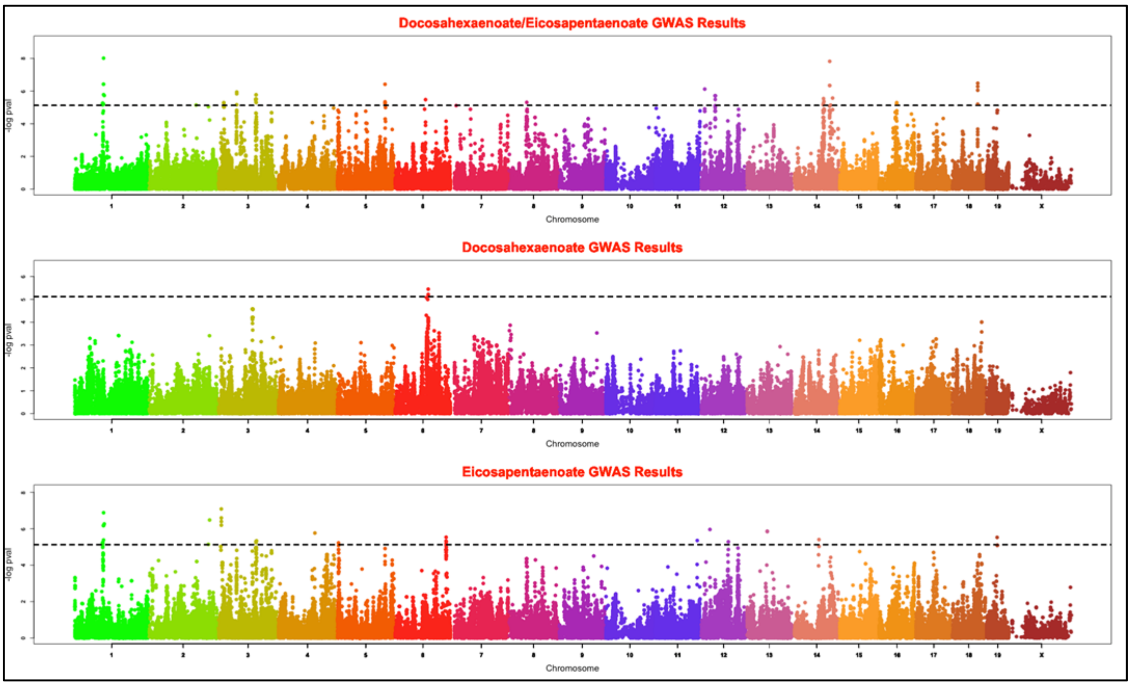
Docosahexaenoate/Eicosapentaenoate GWAS Results
-log pval
Chromosome
Docosahexaenoate GWAS Results
-log pval
Chromosome
Eicosapentaenoate GWAS Results
-log pval
Chromosome

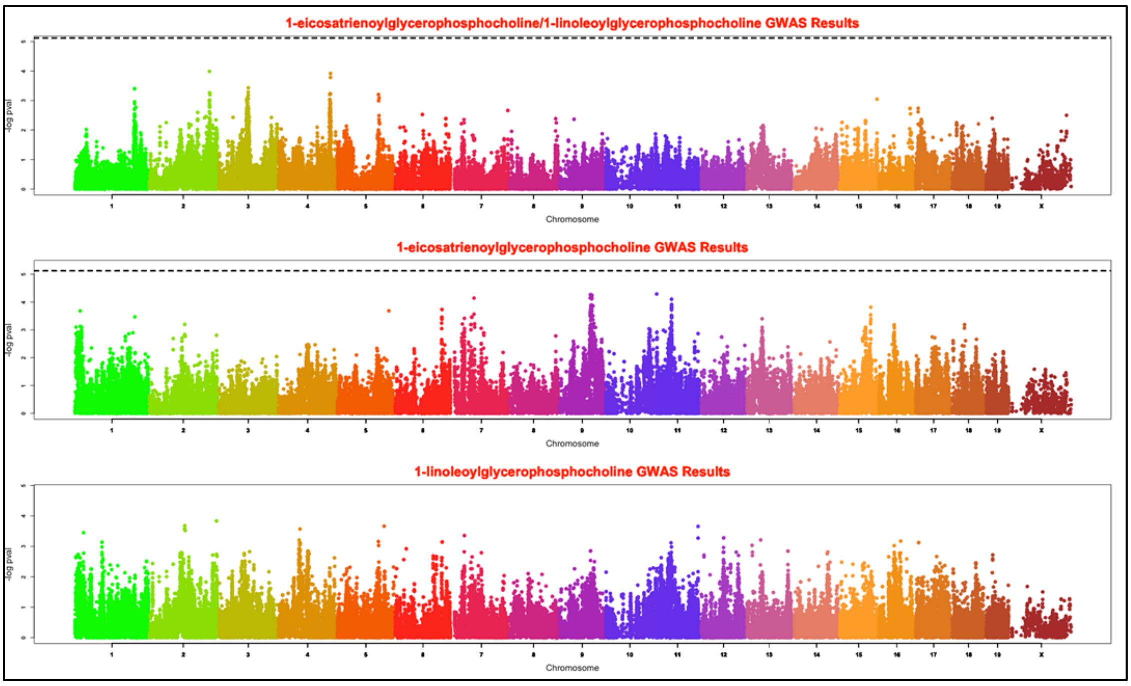
1-eicosatrienoylglycerophosphocholine/1-linoleoylglycerophosphocholine GWAS Results
-log pval
Chromosome
1-eicosatrienoylglycerophosphocholine GWAS Results
-log pval
Chromosome
1-linoleoylglycerophosphocholine GWAS Results
-log pval
Chromosome

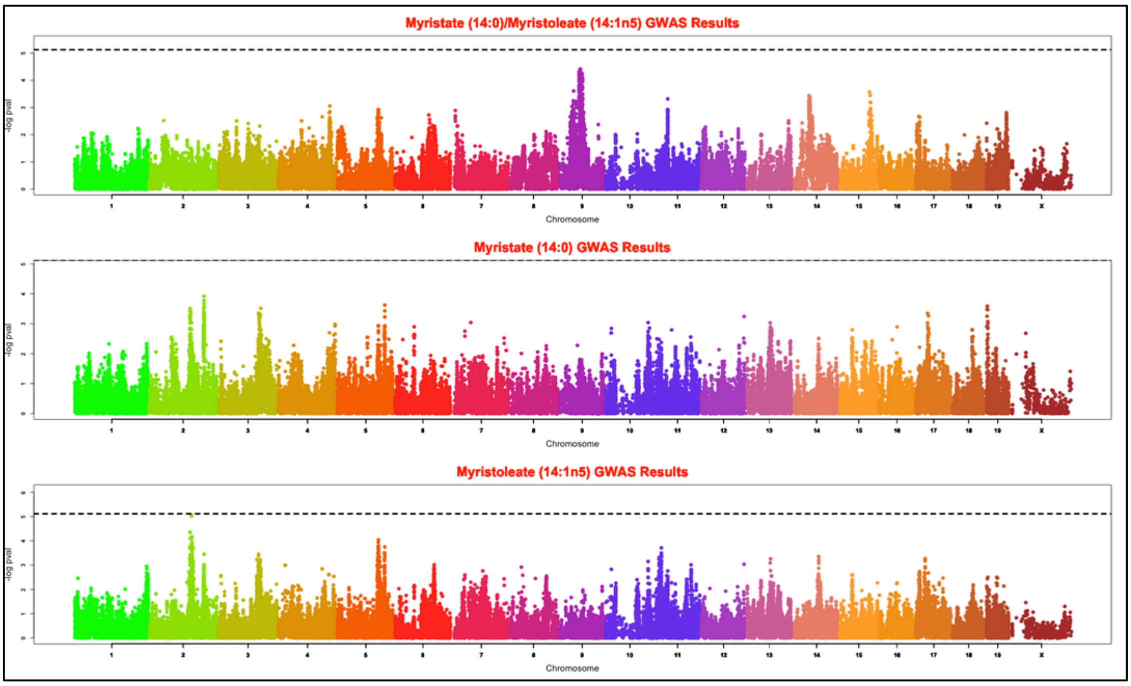
Myristate (14:0)/Myristoleate (14:1n5) GWAS Results
-log pval
Chromosome
Myristate (14:0) GWAS Results
-log pval
Chromosome
Myristoleate (14:1n5) GWAS Results
-log pval
Chromosome

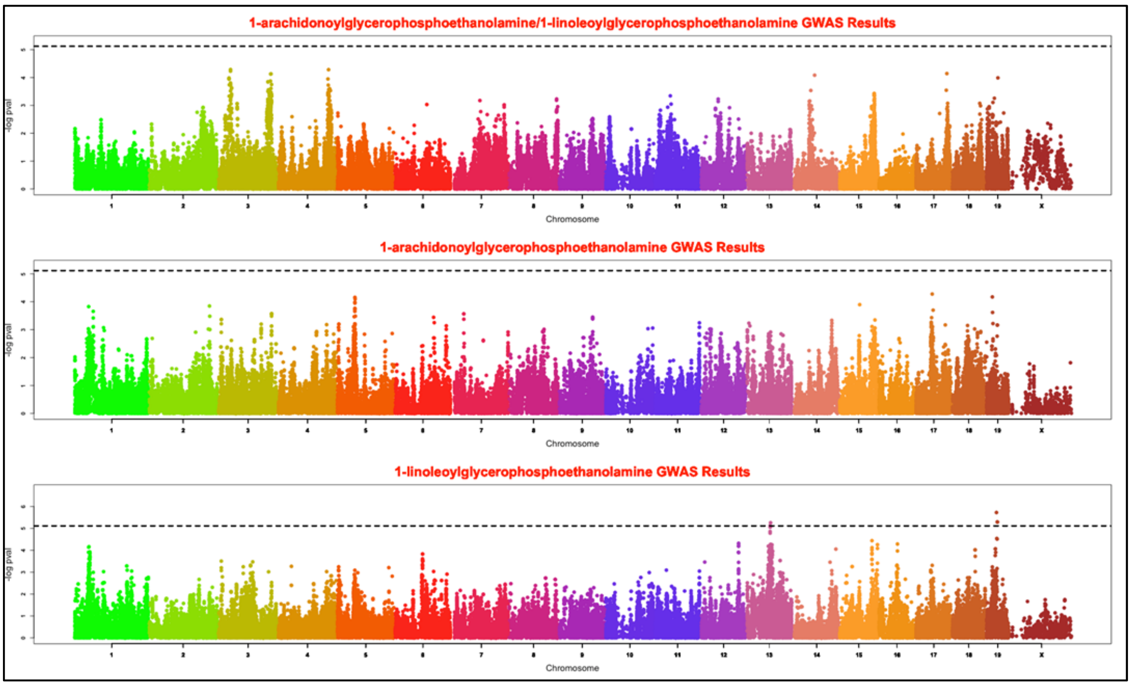
1-arachidonoylglycerophosphoethanolamine/1-linoleoylglycerophosphoethanolamine GWAS Results
-log pval
Chromosome
1-arachidonoylglycerophosphoethanolamine GWAS Results
-log pval
Chromosome
1-linoleoylglycerophosphoethanolamine GWAS Results
-log pval
Chromosome

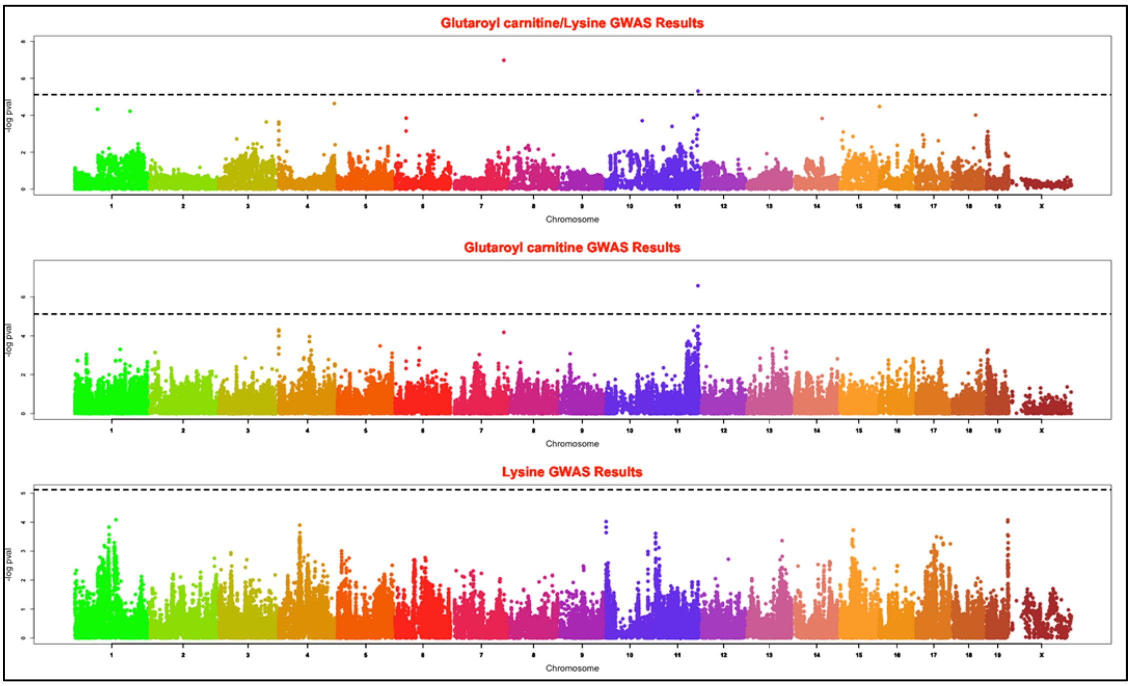
Glutaroyl carnitine/Lysine GWAS Results
-log pval
Chromosome
Glutaroyl carnitine GWAS Results
-log pval
Chromosome
Lysine GWAS Results
-log pval
Chromosome

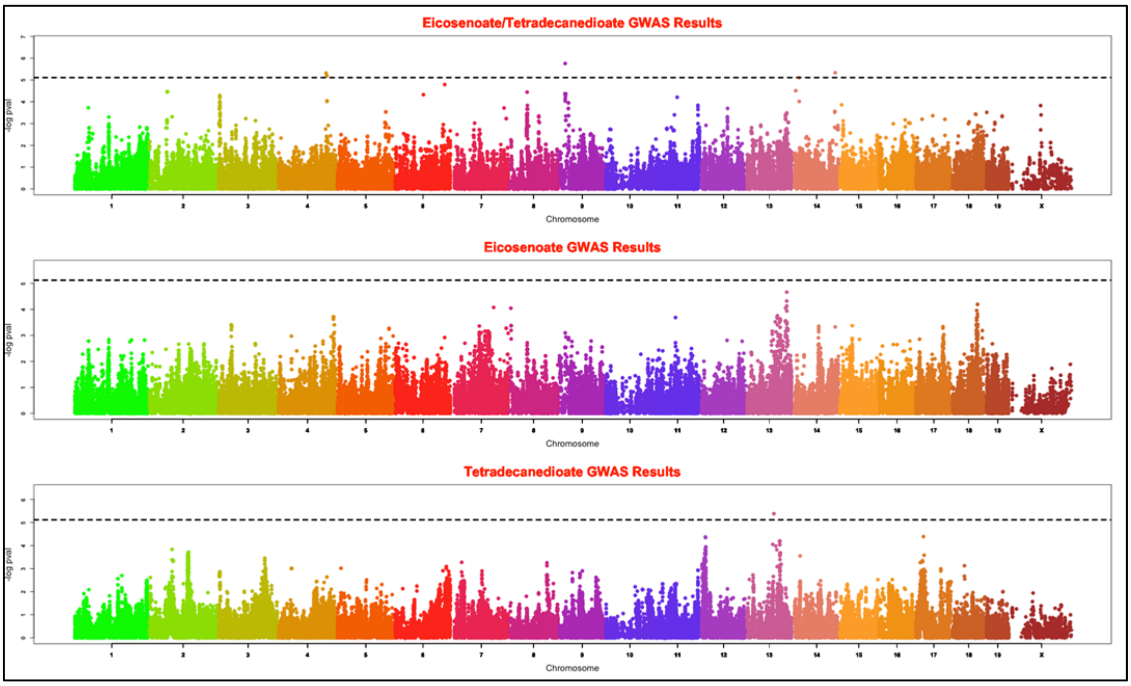
Eicosenoate/Tetradecanedioate GWAS Results
-log pval
Chromosome
Eicosenoate GWAS Results
-log pval
Chromosome
Tetradecanedioate GWAS Results
-log pval
Chromosome
