# Supplementary material for: Genetic regulation of mouse liver metabolite levels
Source: Mol Syst Biol. 2014 May 23;10(5):730. doi: 10.15252/msb.20135004 (PMC4188043; doi:10.15252/msb.20135004)

Figure S4. Metabolite-traits-transcript relationship. Two-dimensional hierarchical clustering and heatmap of correlation between 969 probesets, 58 clinical traits, and 145 metabolites is presented. Metabolites and clinical traits are shown along the x-axis and transcripts along the y-axis. Each metabolite class is represented by color bars below the dendrogram. The color key for metabolites is: olive for Lipids, red for Amino Acids, orange for Peptides, blue for Carbohydrates, brown for Vitamins and Cofactors, green for Energy Metabolism, black for Nucleotides, and grey for Xenobiotics. Clinical traits are represented by the turquoise color bar. Within the heatmap, red represents positive correlation, blue represents negative correlation, and white represents non-significant correlation

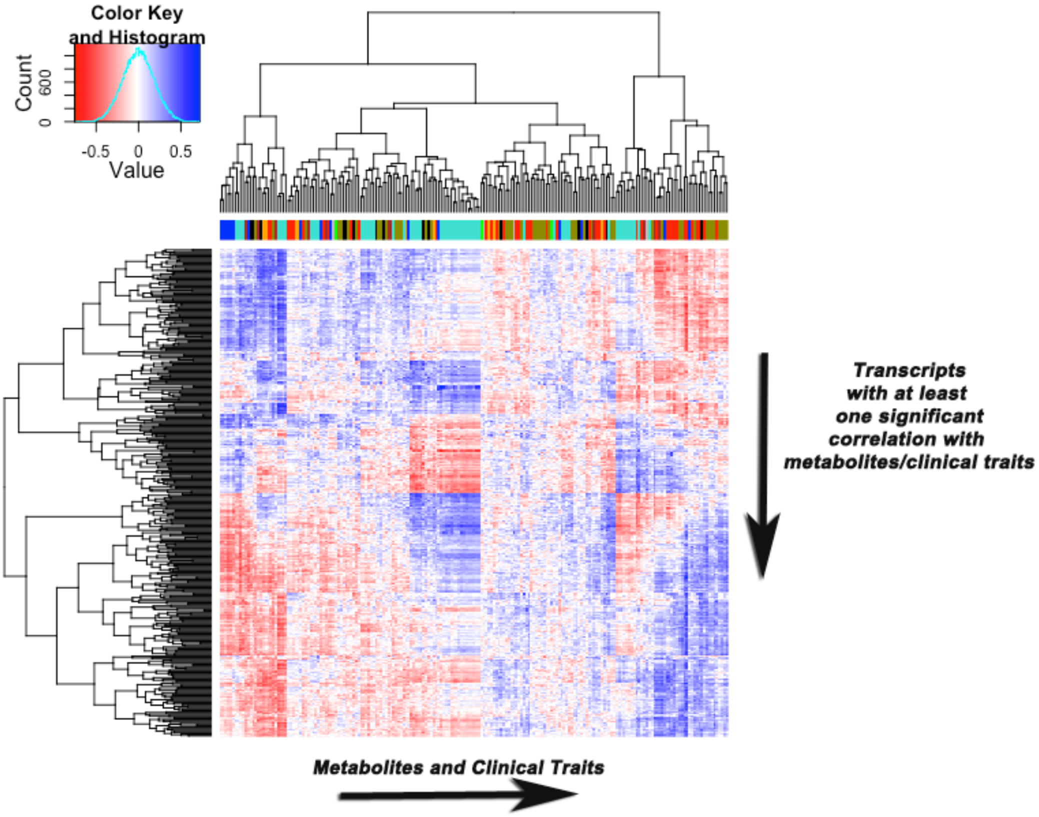

Supplement: Supplementary file 4 — Supplementary Figure S4 [file MSB-10-5-730-s4.pdf]
