# Supplementary material for: Genetic regulation of mouse liver metabolite levels
Source: Mol Syst Biol. 2014 May 23;10(5):730. doi: 10.15252/msb.20135004 (PMC4188043; doi:10.15252/msb.20135004)
Supplement: Supplementary file 12 — Review Process File [file MSB-10-5-730-s12.pdf]

molecular systems biology

Genetic regulation of mouse liver metabolite levels

Anatole Ghazalpour, Brian J. Bennett, Diana Shih, Nam Che, Luz Orozco, Calvin Pan, Raffi Hagopian, Aiqing He, Paul Kayne, Wen-pin Yang, Todd Kirchgessner, Aldons J Lusis

Corresponding author: Anatole Ghazalpour, UCLA

Review timeline:

Submission date:	11 April 2012
Editorial Decision:	16 June 2012
Re-submission:	20 November 2013
Editorial Decision:	10 January 2014
Revision received:	27 February 2014
Editorial Decision:	20 March 2014
Revision received:	04 April 2014
Accepted:	14 April 2014

Editor: Thomas Lemberger

No Peer Review Process File is available with this article, as the authors have chosen not to make the review process public in this case.

© European Molecular Biology Organization
